# Secondary donor-derived humanized CD19-modified CAR-T cells induce remission in relapsed/refractory mixed phenotype acute leukemia after allogeneic hematopoietic stem cell transplantation: a case report

**DOI:** 10.1186/s40364-020-00216-1

**Published:** 2020-08-31

**Authors:** Meng-Yun Li, Zhi-Hong Lin, Ming-Ming Hu, Li-Qing Kang, Xiao-xia Wu, Qi-wei Chen, Xin Kong, Jian Zhang, Hui-Ying Qiu, De-Pei Wu

**Affiliations:** 1 Jiangsu Institute of Hematology, National Clinical Research Center for Hematologic Diseases, Collaborative Innovation Center of Hematology, The First Affiliated Hospital of Soochow University, Institute of Blood and Marrow Transplantation, Soochow University, 188 Shizi Street, Suzhou, 215006 P.R. China; 2 Suzhou Yongding Hospital, Suzhou, People’s Republic of China; 3 Institute of Biomedical Engineering and Technology, Shanghai Engineering Research Center of Molecular Therapeutics and New Drug Development, School of Chemistry and Molecular Engineering, East China Normal University, NO, 3663 North Zhongshan Road, Shanghai, 200065 China; 4 Shanghai Unicar-Therapy Bio-medicine Technology Co., Ltd, No 1525 Minqiang Road, Shanghai, 201612 China

**Keywords:** CD19, Chimeric antigen receptor T cells, Donor-derived, Humanized, Mixed phenotype acute leukemia

## Abstract

**Background:**

Mixed phenotype acute leukemia (MPAL) is a rare leukemia and is regarded as a high-risk entity with a poor prognosis. Induction therapy of an acute lymphoblastic leukemia type or hybrid regimen and hematopoietic stem cell transplantation has been recommended for MPAL. However, the optimal therapies for relapsed or refractory MPAL remain unclear, especially for relapse after stem cell transplantation. Donor-derived chimeric antigen receptor T (CAR-T) cell therapy may be a promising therapeutic option for patients with MPAL who express target antigens and have relapsed after stem cell transplantation. However, recurrence remains a challenge, and reinfusion of CAR-T cells is not always effective. An infusion of secondary donor-derived humanized CD19-modified CAR-T cells may be effective in inducing remission.

**Case presentation:**

We report a case of MPAL with CD19 expression. The patient was treated with acute lymphoblastic leukemia-like induction and consolidation therapies but remained positive for SET-NUP214 fusion gene transcript. He subsequently underwent a haploidentical stem cell transplantation but relapsed within 6 months. He then underwent donor-derived CD19-targeted CAR-T cell therapy and achieved a sustained, complete molecular remission. Unfortunately, he developed a CD19-positive relapse after 2 years. Donor-derived humanized CD19-directed CAR-T cells induced a second complete molecular remission without severe cytokine release syndrome or acute graft-versus-host disease.

**Conclusion:**

This case demonstrated the efficacy and safety of humanized donor-derived CD19-modified CAR-T cell infusion for treating the recurrence of MPAL previously exposed to murine-derived CD19-directed CAR-T cells.

## Background

Mixed phenotype acute leukemia (MPAL) is a rare form of leukemia that constitutes only 2 to 5% of all acute leukemia, in which blasts express a complex phenotype of multiple leukemia markers from both myeloid and lymphoid lineages [1–4]. MPAL may show a B/myeloid (B/My, the most prevalent subtype), T/My, or B/T or B/T/My (a rare subtype) phenotype [5]. MPAL is regarded as a high-risk entity with a poor prognosis, particularly in adults, compared with acute myelocytic leukemia (AML) and acute lymphoblastic leukemia (ALL). Optimal therapy for patients with MPAL remains controversial because of the absence of prospective clinical trials owing to the rarity of this entity. Several studies have supported a potential benefit from the application of an ALL-type or hybrid (blending elements of AML and ALL regimens) induction therapy [6–8]. Hematopoietic stem cell transplantation (HSCT) has been reported as being equally beneficial for MPAL compared with that for other acute leukemias in first or second complete remission (CR) [9–12]. However, relapse, especially after transplantation, remains a major challenge. Novel therapies, including small molecular agent, monoclonal antibody, and chimeric antigen receptor T (CAR-T) cell therapy, may induce another remission and longer survival. We report a case of a patient with MPAL who was previously exposed to donor-derived CD19-CAR-T cells with murine single-chain variable fragment (scFvs) for relapse after haploidentical HSCT and achieved remission with treatment by humanized CD19-CAR-T cells derived from the same donor.

## Case presentation

**Fig. 1:**
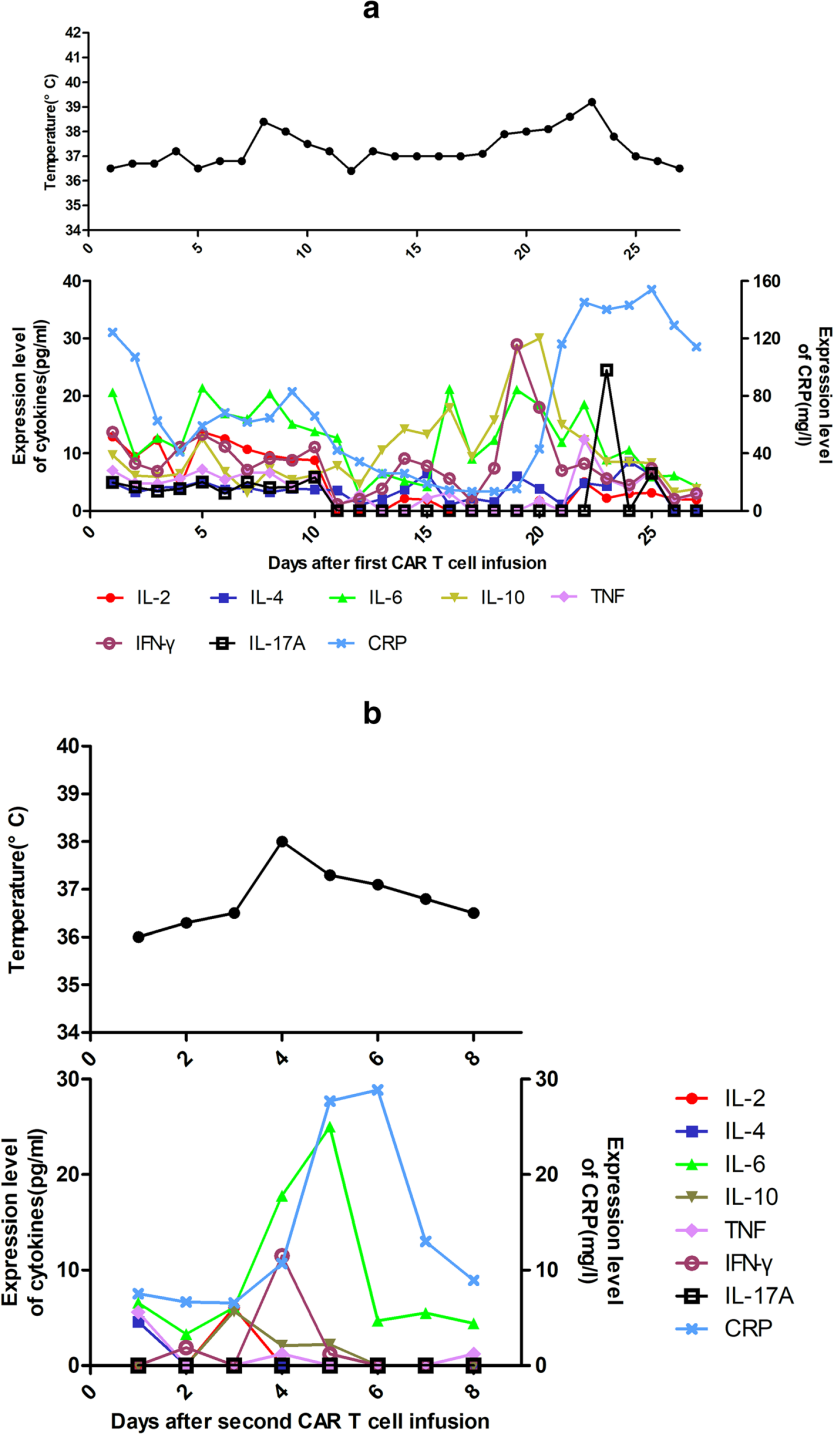
Temperature, serum levels of cytokines and CRP of the patient after CAR T cells infusions. **a** After the first CAR T cells infusion. **b** After the second CAR T cells infusion

**Fig. 2:**
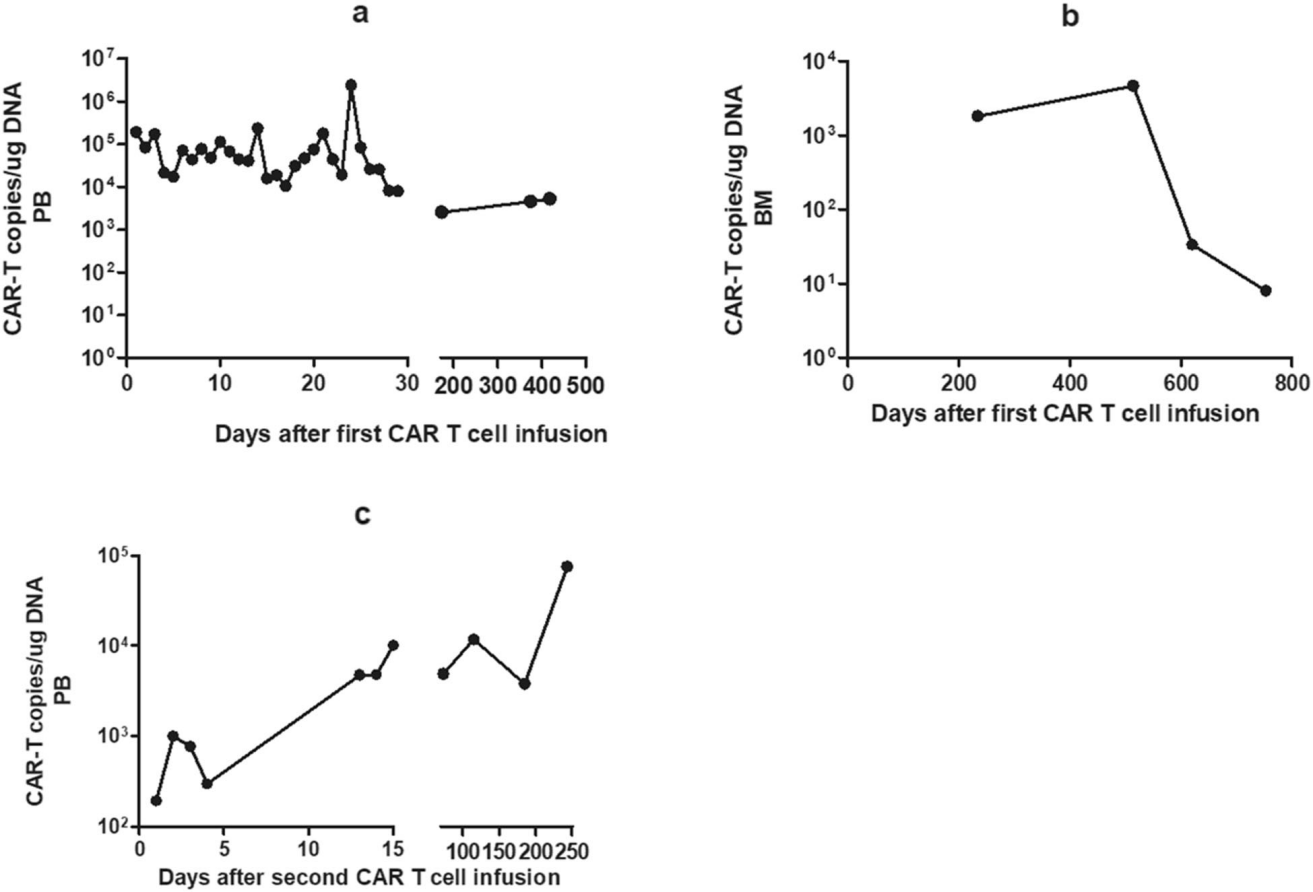
Copies of CD19-specific CAR-T cells in the peripheral blood/bone marrow of the patient after CAR-T cells infusions. Abbreviation: PB, peripheral blood; BM, bone marrow. **a**. CD19-CAR T cell copies from peripheral blood mononuclear cells after the first infusion. **b**. CD19-CAR-T cell copies from bone marrow mononuclear cells after the first infusion. **c**.CD19-CAR-T cell copies from peripheral blood mononuclear cells after the second infusion

A 29 year-old man presented to our hospital with abdominal pain and fever. Peripheral blood examination results revealed a white blood cell count of 0.56 × 10^9^/L, a hemoglobin level of 7.7 g/dl, and a platelet count of 43 × 10^9^/L. Bone marrow examination revealed an increase of blasts up to 67.2% and showed negative results for myeloperoxidase staining. Flow cytometry analysis demonstrated an abnormal blast population (48%) expressing CD7, CD34, HLA-DR, CD10, CD19, CD33, CD117, cCD79a, and cCD3. Cytogenetic and molecular biology studies showed an abnormality of 46,XY,add(6)t(?1;6)(?p31;p24),del (16)(?q11)[6]/46,XY[4] and SET-NUP214 fusion gene transcript. No gene mutation was detected by next-generation DNA sequencing. These findings indicated a diagnosis of B/T MPAL with myeloid lineage expression. Induction chemotherapy with idarubicin, vincristine, and dexamethasone achieved morphological CR. Subsequently, we gave the patient consolidated treatments, including one cycle of pegaspargase combined with hyper-CVAD-B regimen (with high-dose cytarabine and methotrexate) and one cycle of hyper-CVAD-A regimen (with cyclophosphamide, pharmorubicin, vincristine, and dexamethasone). After treatment, the patient achieved a sustained CR, although he remained positive for the SET-NUP214 transcript as detected by quantitative real-time polymerase chain reaction. He subsequently underwent a haploidentical stem cell transplantation from his father after conditioning with the busulfan/cyclophosphamide regimen. Peripheral myeloid engraftment (absolute neutrophil count, > 0.5 × 10^9^/L) was evident on day 12, and he was platelet transfusion-dependent (platelet count, > 50 × 10^9^/L) until day 14 post-HSCT. The patient sustained a complete molecular remission (CMR) until 6 months later. He then exhibited a recurrence of leukemia with the appearance of blasts (48%) in bone marrow aspirates and a positive minimal residual disease (MRD) (85.1%) with a phenotype of B-ALL (with expression of CD7, CD19, CD33, and cCD79a). He was positive for the SET-NUP214 transcript at the time of relapse. Chimerism analysis using multiplex polymerase chain reaction to amplify an informative short tandem repeat demonstrated 51.4% donor cells. CAR-T cell therapy targeting CD19 derived from the same donor was then conducted. The CD19-directed CAR-T cells carried scFvs derived from murine antibodies. The patient underwent a fludarabine (30 mg/m^2^, days 1–3) and cyclophosphamide (300 mg/m^2^, days 1–3) based lympho-depletion regimen before CAR-T cell infusion. The total dose was 5 × 10^6^ per kg of body weight CAR-positive T cells (transduction efficiency, 34.49%). The patient developed moderate fever twice, on days 5 and 17 after infusion, which lasted 2 and 5 days, respectively. There were no hypotension, tachycardia, hypoxia, fatigue, or life-threatening events, such as disseminated intravascular coagulation or multi-organ dysfunction after transfusion. After treatment with nonsteroidal drugs and anti-infection therapy, the patient’s body temperature returned to a normal level. Additionally, a series of PB cytokines, including interleukin (IL)-2, IL-4, IL-6, IL-10, tumor necrosis factor, interferon-γ, and IL-17A levels, showed no significant increase; only C-reactive protein (CRP) showed a maximum fold change of 10 on day 22 (Fig. 1a). Generally, the patient’s cytokine release syndrome (CRS) was grade 2. Bone marrow examination suggested CMR 9 days after CAR-T cell infusion, and the patient maintained a state of CMR combined with persistence of CD19-directed CAR-T cells for 20 months without obvious development of graft-versus-host disease (GvHD) (Fig. 2, a and b). However, quantitative real-time polymerase chain reaction analysis detected that he was positive for SET-NUP214 transcript 20 months after CAR-T cell infusion. Meanwhile, a sharp decrease in bone marrow DNA copies of anti-CD19 CAR-T cells occurred (33.4 copies per microgram) (Fig. 2b). Nonetheless, the patient still attained morphological remission and was negative for MRD detected by flow cytometry analysis (0.0 × 10^− 4^), with complete donor chimerism. Unfortunately, the patient did not undergo a bone marrow examination again during follow-up. Five months later, morphological relapse occurred with 38% blast cells. A positive MRD (27.8%) and SET-NUP214 transcript (231.15%) were detected, accompanied by an extremely low number of DNA copies of anti-CD19 CAR-T cells (8.05 copies per microgram) in the bone marrow specimen (Fig. 2b) and 68.7% donor cell chimerism. The patient was again treated with idarubicin, vincristine, and dexamethasone as a reinduction chemotherapy. However, flow cytometry analysis revealed 45.5% of blast cells with a phenotype of B-ALL (with expression of CD7, CD34, HLA-DR, CD19, CD33, CD38, cCD79a, CD71, and CD22) after chemotherapy, and the bone marrow short tandem repeat dropped to 55.5%. The patient received a second infusion of donor-derived humanized CD19-directed CAR-T cells, which has the same costimulatory domain and coactivated domain as mouse-derived CAR-19, except that in the scFv domain of CAR, the humanized CAR-19 recognition site is HD37.The total dosage of humanized CD19-directed CAR-T cells was 1 × 10^7^/kg (transduction efficiency 42%). The same lympho-depletion regimen of FC (fludarabine /cyclophosphamide) conducted before CAR-T cell infusion was administered. The patient developed a temporary low fever on the first day after infusion, for which nonsteroidal anti-inflammatory drugs were effective. Cytokine and CRP levels increased slightly, indicating grade 1 CRS (Fig. 1b). The level and duration of fever and the increase in cytokine and CRP levels were lower than the previously observed degree after the first CAR-T cell infusion. Moreover, new-onset acute GvHD did not occur after the second CAR-T cell infusion. CR, 99% donor chimerism, and significantly increased expansion and persistence of CD19-directed CAR-T cells (Fig. 2c) were achieved after infusion. The patient was still alive at the time of the 8 month follow-up evaluation.

## Discussion

MPAL is considered a high-risk leukemia with a dismal prognosis, and patients should be considered candidates for post-remission consolidation with HSCT, in either CR1 or CR2, particularly patients who are consecutively positive for MRD [9–12]. Patients with MPAL who are undergoing HSCT might achieve similar results compared with those with AML or ALL; however, a population of patients experience relapse after HSCT. Post-HSCT relapse is generally associated with a grave prognosis and is the primary cause of death for many of these patients. Promising clinical outcomes have been reported in refractory or relapsed B-cell malignancies, including patients with ALL and diffuse large B-cell lymphoma treated with CD19-targeted CAR-T cells [13–17]. Compared with autologous CAR-T cell therapy, allogeneic CAR-T cells have more advantages. They are free of contamination from inadvertently transduced leukemic blasts, have a lower incidence of T cell dysfunction, are easier to harvest, and are more tolerated of ex vivo manipulation. Moreover, allogeneic CAR-T cells can be prepared in advance and kept in reserve for when they are needed, reducing the waiting time for relapsed patients. Donor-derived CD19-targeted CAR-T cell therapy might be a promising therapeutic option for relapsed or refractory MPAL after HSCT, as more than two-thirds of patients with MPAL express B-lineage antigens on blasts. However, durable remission after CD19-targeted immunotherapy remains a major challenge. A large proportion of CR patients relapse within 1 year [18, 19]. Several assumptions have been made regarding the causes of recurrence, including depletion of CAR-T cells, abnormal function of CAR-T cells induced by the microenvironment or other factors, and a loss or diminished expression of targeting antigens on the cell surface [20–25].

In our case, a patient with a consecutively positive MRD pre-HSCT quickly relapsed within 6 months after HSCT. He then successfully achieved CMR after CD19-targeted CAR-T cell therapy derived from the same donor. Unfortunately, he relapsed again when extremely low numbers of DNA copies of CD19-modified CAR-T cells were detected in his bone marrow 2 years after the first infusion, and the blasts remained CD19-positive. Obviously, although CAR-T cells persisted for a relatively long time in this case, CAR-T cell depletion eventually occurred. However, the question of how to extend CAR-T cell persistence in vivo has still not been answered satisfactorily, and there is no optimal treatment guideline for recurrence after CD19-modified CAR-T cell therapy. Recent studies have confirmed that the host’s immune response can recognize epitopes of murine scFv domains of the previous CAR structure, resulting in invalidation of subsequent infusions [26]. Therefore, we chose to treat donor-derived humanized CD19 CAR-T cells and induced a second CMR without severe CRS and acute GvHD. Additionally, the patient maintained continuous CMR, and the persistence of CD19-directed CAR-T cells was detected during the 8 month follow-up period. To address the possible causes of recurrence after CAR-T cell treatment and to improve long-term outcome, a cohort of studies and clinical trials have been conducted. These involved dual-signaling CAR-T cell therapies and preventive infusion of a second CAR-T cell product targeting a different antigen when a significant and sustained decline of copies of previous CAR-T cells was detected before the possibility of relapse [27–29].

## Conclusion

Our case demonstrates the efficacy and safety of humanized donor-derived CD19-modified CAR-T cells infusion for treating recurrent MPAL, which was previously exposed to murine-derived CD19-CAR-T cells. Further verification is needed to evaluate this strategy.

## Data Availability

Data sharing is not applicable to this article as no datasets were generated or analysed during the current study.

## Abbreviations

MPAL: mixed phenotype acute leukemia
ALL: acute lymphoblastic leukemia
AML: acute myelocytic leukemia
HSCT: hematopoietic stem cell transplantation
CAR-T: chimeric antigen receptor T
scFvs: single-chain variable fragment
CR: complete remission
CMR: complete molecular remission
MRD: minimal residual disease
CRP: C-reactive protein
CRS: cytokine release syndrome
GvHD: graft-versus-host disease
